# Typhus Fever in Mexico

**Published:** 1893-01

**Authors:** 


TYPHUS FEVER is prevailing extensively in Northern Mexico. It is reported to have crossed over into Texas and made its appearance at El Paso, Laredo and Eagle Pass. At the latter place, it is said that H. Ornelas, brother of the Mexican Consul Dr. Ornelas, at San Antonio, is down with the fever. State Health Officer Swearingen, some weeks ago, put Dr. Yandell, State Quarantine Officer at El Paso, on duty, and an inspection service was at once instituted. On receipt of news that the fever had reached Eagle Pass, State Quarantine Officer, M. K. Lott was at once, by wire, re-assigned to duty, and a rigid inspection of all incoming passengers was begun. The Texas health authorities are wide-awake, and will keep out all infectious diseases. Everything is in readiness, at every gate of travel, all around the State of Texas, to put on inspection service at a minute's notice. When the cholera scare at Little Rock, Ark., occurred, Texas was ready, by touching a button (as it were) in the State Health Officer's office, to clap on the brakes, and inspect all comers into the State; but it was not necessary.

